# Evidence of Enriched, Hadean Mantle Reservoir from 4.2-4.0 Ga zircon xenocrysts from Paleoarchean TTGs of the Singhbhum Craton, Eastern India

**DOI:** 10.1038/s41598-018-25494-6

**Published:** 2018-05-04

**Authors:** Trisrota Chaudhuri, Yusheng Wan, Rajat Mazumder, Mingzhu Ma, Dunyi Liu

**Affiliations:** 1 Department of Geology, University of Calcutta, 35, Ballygunge Circular Road, Kolkata, 700019 India; 2 Beijing SHRIMP Center, Institute of Geology, Chinese Academy of Geological Sciences, Beijing, 100037 China; 3 Department of Applied Geology, Faculty of Engineering and Science, Curtin University Malaysia, CDT 250, Miri, 98009 Sarawak, Malaysia

## Abstract

Sensitive High-Resolution Ion Microprobe (SHRIMP) U-Pb analyses of zircons from Paleoarchean (~3.4 Ga) tonalite-gneiss called the Older Metamorphic Tonalitic Gneiss (OMTG) from the Champua area of the Singhbhum Craton, India, reveal 4.24-4.03 Ga xenocrystic zircons, suggesting that the OMTG records the hitherto unknown oldest precursor of Hadean age reported in India. Hf isotopic analyses of the Hadean xenocrysts yield unradiogenic ^176^Hf/^177^Hf^initial^ compositions (0.27995 ± 0.0009 to 0.28001 ± 0.0007; ɛHf[t] = −2.5 to −5.2) indicating that an enriched reservoir existed during Hadean eon in the Singhbhum cratonic mantle. Time integrated ɛHf[t] compositional array of the Hadean xenocrysts indicates a mafic protolith with ^176^Lu/^177^Hf ratio of ∼0.019 that was reworked during ∼4.2-4.0 Ga. This also suggests that separation of such an enriched reservoir from chondritic mantle took place at 4.5 ± 0.19 Ga. However, more radiogenic yet subchondritic compositions of ∼3.67 Ga (average ^176^Hf/^177^Hf^initial^ 0.28024 ± 0.00007) and ~3.4 Ga zircons (average ^176^Hf/^177^Hf^initial^ = 0.28053 ± 0.00003) from the same OMTG samples and two other Paleoarchean TTGs dated at ~3.4 Ga and ~3.3 Ga (average ^176^Hf/^177^Hf^initial^ is 0.28057 ± 0.00008 and 0.28060 ± 0.00003), respectively, corroborate that the enriched Hadean reservoir subsequently underwent mixing with mantle-derived juvenile magma during the Eo-Paleoarchean.

## Introduction

Zircons, which are the only representatives of the oldest rocks on Earth, preserve robust records of chemical and isotopic characteristics as well as the history of the generation of their parent rocks^1–5^. Thus far, the oldest recorded rocks on Earth are the 4.03-3.92 Ga gneisses from the Acasta Gneiss Complex, Slave Province, Canada^1–7^. Zircons older than these have been found as detrital grains within metasedimentary rocks from Western Australia^8–13^, Western Tibet^14^, Brazil^15^ and Southern China^16^ and as xenocrysts within meta-igneous rocks from Western Australia^17^ and Central China^18^. The examination of the Hf isotopic compositions of the oldest zircons, can further contribute to a comprehensive understanding of the differentiation of the early silicate Earth^3,19–22^. Contrasting isotopic signatures and interpretations from such databases have fueled a persistent debate about the nature of the source reservoir of the protolith generating Hadean and Eoarchean zircons and the composition of the earliest crust that developed during the Hadean^3,6,12,19–21^. Hf isotopes are tracers that are very widely used to explicate crustal generation processes^19–21^. Previously, Lu-Hf isotopic data from the Jack Hill’s zircons of Hadean age revealed both supra-chondritic and subchondritic initial ^176^Hf/^177^Hf values^23^. Highly radiogenic Hadean and Eoarchean zircons reported from the Jack Hill’s metaconglomerate^20,23^ implied the presence of depleted reservoirs, which was in contradiction with concurrent Pb-Pb and Lu-Hf isotopic studies yielding predominantly unradiogenic ɛHf[t] values^19,21,24^. The highly positive values observed in previous studies were later described as the artifacts of isotopic mixing between different age domains of zircons with very fine oscillatory zoning^25^. Here, we report xenocrystic zircons of Hadean (~4.0-4.2 Ga) to Eoarchean age (~3.7 Ga) from the Paleoarchean (~3.4 Ga) Tonalite-Trondhjemite-Granodiorite gneisses (TTG), called the Older Metamorphic Tonalitic Gneiss (OMTG), of the Paleo-Mesoarchean Singhbhum Craton of Eastern India and confirm that the OMTG holds the hitherto oldest precursor rock recorded in India. We also present Lu-Hf isotopic data from these xenocryst cores and their host zircons that add new information to the Hadean zircon isotopic data repository, augmented with interpretations about the nature of their mantle source and the history of crustal formation events in this craton. In addition, the combined U-Pb and Lu-Hf isotopic data of zircons from two other Paleoarchean TTGs (∼3.4 Ga and ∼3.3 Ga) from different locations within the same craton are also presented to further elucidate the characteristics and heterogeneity of the corresponding mantle source reservoir during the Paleoarchean.

## Geology of Paleoarchean TTGs, Singhbhum Craton

**Figure 1:**
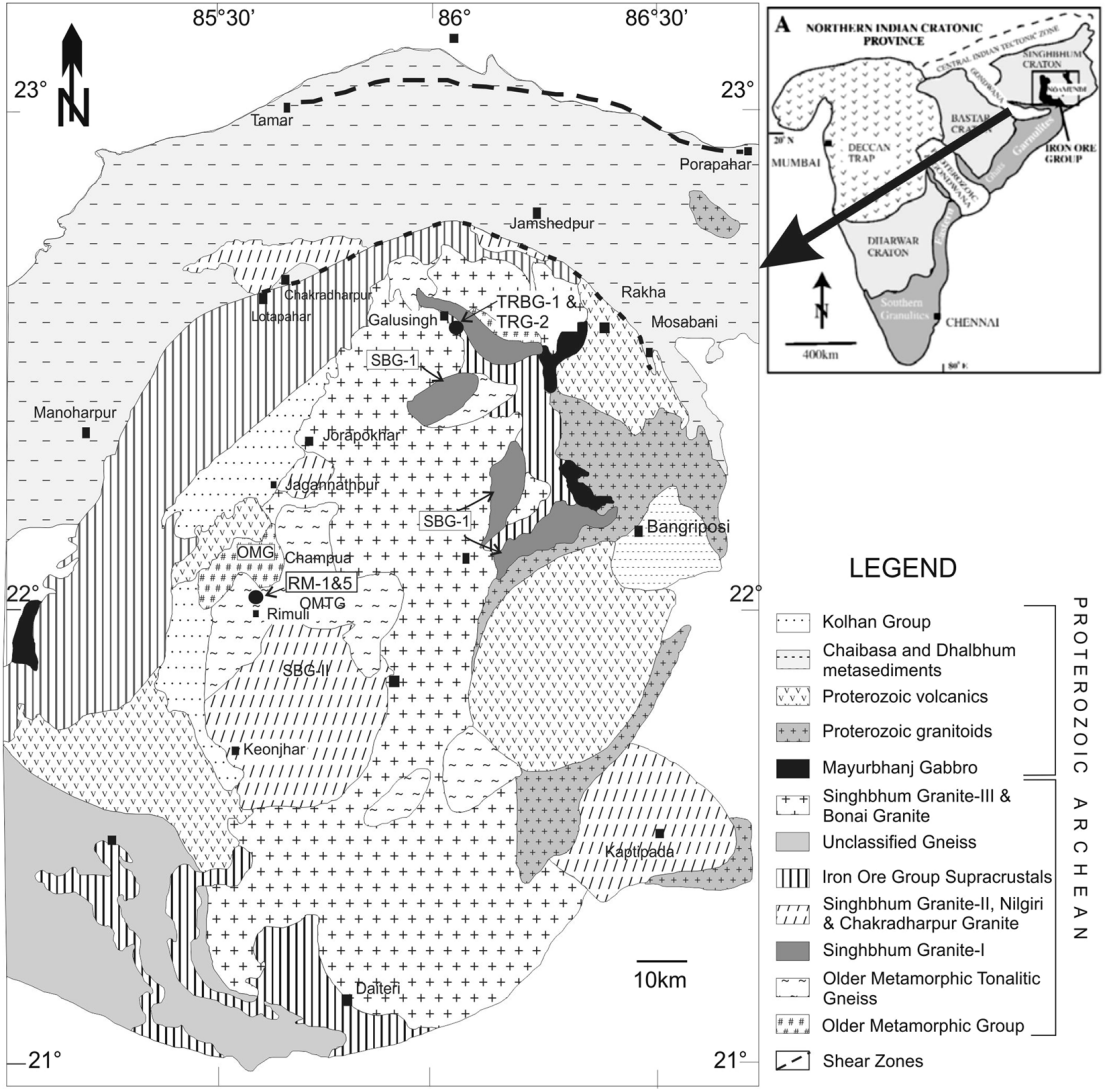
Geological map of the Singhbhum Craton (modified after Upadhyay *et al*.^37^) showing sample locations of the dated TTGs. The map was modified using software Corel Draw^®^ Graphics Suite X7.

The Paleoarchean Singhbhum Craton, India, consists of an Archean nucleus of voluminous TTG gneisses and intrusive granitoids of ∼3.5-3.2 Ga age, flanked by three Paleoarchean greenstone successions, which are named the Iron Ore Group (IOGs)^26–28^ (Fig. 1). The Archean nucleus of this craton is unconformably overlain by Paleoproterozoic supracrustals^27–29^. The Older Metamorphic Group (OMG) comprises interlayered metabasalt (amphibolite) and metasedimentary rocks (biotite-muscovite ± sillimanite ± garnet schists, quartz ± magnetite ± cummingtonite schist; quartz-sericite schist, quartzites and calc-silicates)^26,27,30,31^. The oldest age of the OMG is constrained by a ^207^Pb/^206^Pb ion microprobe age of ∼3.5 Ga, obtained from detrital zircon from quartzites from the Champua area^30,31^. However, the presence of even older inherited cores of ∼3.55-3.6 Ga within these zircons has led authors to suggest that older crust, with a minimum age of ∼3.55-3.6 Ga, existed in the Singhbhum Craton^30,31^. The Sm-Nd isochron age of 3.3 Ga derived from the OMG amphibolites represents their metamorphic age^32^. The Older Metamorphic Tonalitic Gneiss (OMTG) consists of thinly compositionally layered, medium-grained tonalitic to granodioritic gneisses^26,28,33^. According to Hofmann and Mazumder^28^, the OMTG represents a suite of TTGs that formed over an extended period between 3.53-3.45 Ga, whereas the OMG represents a supracrustal assemblage that formed as a greenstone succession. The oldest age obtained from the OMTG is a whole-rock Sm-Nd isochron age of 3775 ± 89 Ma^34^. This age was later questioned and subsequently amended by Moorbath *et al*.^35^ to be closer to 3.4 Ga. However, other older ages recently reported from the OMTG include an age of 3664 ± 79 Ma, which was derived from a whole-rock Pb-Pb isochron^36^, and a xenocrystic zircon core age of ~3.61 Ga (^207^Pb/^206^Pb *in situ* LA-ICP MS dating), which was found within a ~3.4 Ga zircon^37^. Acharyya *et al*.^38^ reported a discordia upper intercept U-Pb zircon age of 3527 ± 17 Ma for the OMTG. Interestingly, the largest population of ^207^Pb/^206^Pb zircon ages of the OMTG from previous studies centered around ~3.4 Ga^33,37–39^, reflecting a major felsic magmatic event. However, the ∼3.6 Ga xenocrysts from the OMTG^37^ and OMG quartzites^30^ indicate that felsic crustal formation was initiated in the Singhbhum Craton well before the major phase of emplacement of the OMTG.

A voluminous TTG (previously named as Singhbhum Granite-I or SG-I^26^)-granitoid suite (SG-II and III^26^), was emplaced in two phases; the older emplacement age (3.45-3.44 Ga^37^) broadly coincides with the emplacement age of the OMTG. The latter phase of emplacement is constrained at approximately 3.35-3.32 Ga^37^. The SG batholith is composite in nature and comprises biotite-granodiorite/granite, adamellite-granite, tonalite and trondhjemite^26,27^. The SG batholith is encircled by three distinct Archean greenstone successions, namely, the eastern, western and southern Iron Ore Group^40–42^ (IOG; Fig. 1). The SHRIMP U-Pb zircon ages of 3507 ± 2 Ma of dacitic lava from the southern IOG^42^ and ~3400 Ma from a tuff layer in the western IOG^41^ confirm the Paleoarchean ages of these greenstone successions. Nelson *et al*.^33^ speculated that the eastern IOG formed between 3.28-3.33 Ga, although its depositional age is still currently unknown.

In this study, zircons from two OMTG samples located to the south of Champua, which is the type locality area of the OMG and OMTG^26,30,31^ (Fig. 1), were investigated. Samples RM-1 and RM-5 were collected from ∼1.5 Km Northwest (21°58′2.8″N, 85°35′53.6″E) and ∼4 Km North (22°00′1.1″N, 85°37′33.3″E) of Rimuli village, respectively (Supplementary Figure SF1). These rocks occur as small, patchy exposures of granite gneiss (OMTG) within a terrain dominated by the OMTG, which contains abundant enclaves of the OMG amphibolite (Supplementary Figure SF3B). These are medium-grained, mesocratic, partially weathered TTG gneisses displaying thin (5–10 mm) compositional banding (Supplementary Figure SF3A). The mesocratic bands comprise medium-grained (1–5 mm) quartz, potassic feldspar, plagioclase and muscovite, whereas the darker bands are mostly comprised of biotite and minor amphibole. Petrographic descriptions of RM-1 and RM-5, as well as their average modal mineralogies, are summarized in Supplementary File SF4.

A sample of coarse-grained, mesocratic granite gneiss (sample TRBG-1; 22°32′43″N, 86°05′53″E; Fig. 1) was collected from the older phase (phase-I) of the Singhbhum Granite (SG-I of the Rajnagar-Kuyali sector^26^; Supplementary Figure SF2), adjacent to the metabasalt-quartzite sequence of the Eastern IOG greenstone belt to the southeast of Galusingh. A relatively fresh, mesocratic to leucocratic, medium- to coarse-grained, equigranular granite (sample TRG-2; 22°32′54″N, 86°04′52″E) corresponding to the latest phase (phase-III) of the Singhbhum Granite (SG-III^26^; Supplementary Figure SF2), intrudes both the metabasalt-fuchsite quartzite-chert association of the Eastern Iron Ore Group (EIOG) greenstone belt and the TRBG-1, near sample collection site of TRBG-1. This granite is crudely foliated and contains enclaves of TRBG-1 (or SBG-I; Supplementary Figure SF3C) and the amphibole schist of the EIOG greenstone belt, suggesting that it is younger than both the granite gneiss (TRBG-I) and the EIOG.

## Results

### Description of zircons and U-Pb data

**Figure 2:**
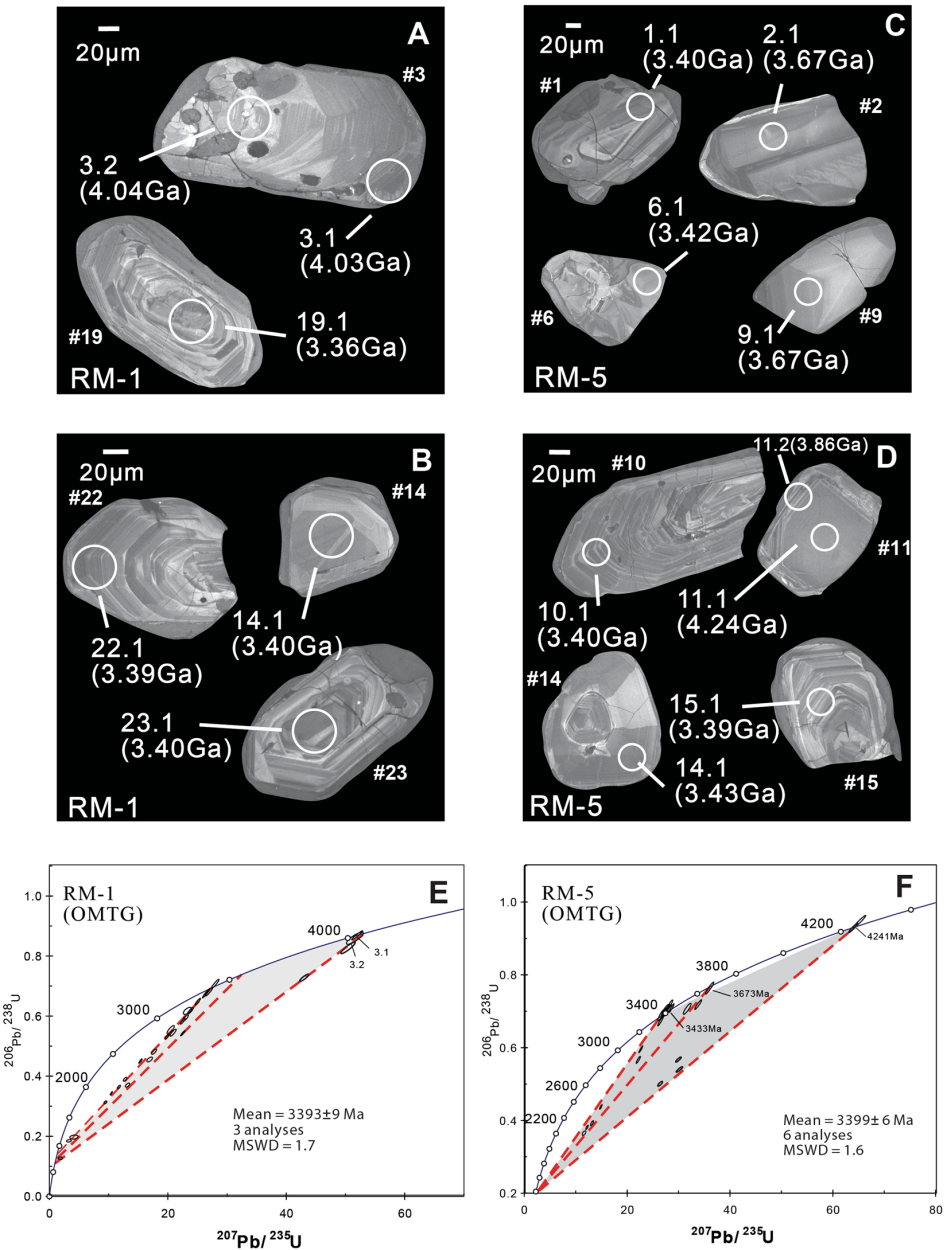
(**A**,**B**) Cathodoluminiscence (CL) image of zircons from OMTG sample RM-1, (**C**,**D**) from sample RM-5. Circles represent analyses spot positions with spot numbers and their ages in Ga. Grain numbers are shown beside each grain (e.g. #1). (**E**,**F**) U-Pb concordia plots for the zircons from samples RM-1 and RM-5 respectively (ages represent weighted mean of ages in Ma). Separate discordia lines were fitted through different age groups of zircons. Error ellipses are shown at 1σ.

The ^207^Pb/^206^Pb age data of 85 points from 23 zircon grains were obtained from four samples of the Older Metamorphic Tonalitic Gneiss (OMTG; RM-1 & 5), Singhbhum Granite Phase-I (SG-I; sample TRBG-1) and Singhbhum Granite Phase-III (SG-III; sample TRG-2); these data are presented in Table 1 and Supplementary Table S1. Two zircon grains from RM-1 (grain #3) and RM-5 (grain #11) exhibit significantly older ages (Hadean) than the rest of the analyzed grains (Eoarchean to Paleoarchean) in this study. Grain #3 is subhedral and displays oscillatory zoning in the cathodoluminescence (CL) image (Fig. 2A); it yields three analyses with ^207^Pb-^206^Pb concordant ages of 4031 ± 5, 4036 ± 15, and 4057 ± 8 Ma (Table 1; Supplementary Table S1). A second, relatively smaller, subhedral grain (grain #11) shows a homogenous core in its CL image and yields two concordant ages of 4241 ± 4 and 4239 ± 4 Ma (Table 1), while its rim shows thin oscillatory zoning (Fig. 2D) and yields discordant ages of ~3.8-3.9 Ga (Supplementary Table S1). The differences in age and Hf isotopic compositions between these two grains and the rest of the zircon population, combined with their subhedral grain shapes, indicate that these Hadean zircons are inherited in origin^43^. The oscillatory zoning and higher Th/U ratios (0.44-0.65) of the Hadean zircons suggest an igneous origin^44^, although exceptions can occur^45^. Three analyses from the oscillatory zoned rim of an old xenocrystic core with an age of 4241 ± 4 Ga (grain #11) from sample RM-5 yield >10% discordance, thus reflecting Pb loss, which implies hat this rim is probably older than ~3.8-3.9 Ga. In sample RM-5, grain #2 exhibits an inherited core with a concordant age of 3670 ± 7 Ma, which is homogenous in its CL image (Fig. 2C) and is surrounded by an oscillatory zoned growth rim. Another xenocryst from the same sample (grain #9), which has resorbed grain boundaries and broad, faint zoning visible in CL image, yields a concordant age of 3673 ± 7 Ma (Fig. 2C,F). Another older age spot in zircon from sample RM-5 (19.1) yields a concordant age of 3595 ± 12 Ma. The U-Pb analyses of the RM-1 and RM-5 zircons yield ^207^Pb/^206^Pb age data that define linear arrays, yielding concordia intercepts at ages of 3393 ± 9 Ma (MSWD = 1.7; n = 3) and 3399 ± 6 Ma (MSWD = 1.6; n = 6), respectively (Fig. 2E,F). Most of the dated zircons exhibit regular oscillatory zoning from core to rim (Fig. 2A–D). Some grains exhibit homogenous cores surrounded by growth-zoned rims (Fig. 2B; grain #23; sample RM-1) but yield a consistent age of ∼3.4 Ga (spot 23.1; Table-1). The lower intercept ages of RM-1 and RM-5 are ∼900 and ∼1200 Ma, respectively which broadly coincide with a ∼1.2-1.0 Ga magmatic event related to the late phase of regional dyke swarm emplacement known as the ‘Newer Dolerite Dykes’^26^. Zircons from samples RM-1 and 5 are euhedral to subhedral, and the presence of irregular boundaries in some grains can be attributed to solid-state recrystallization^46^. The ~4.0 Ga zircon grain #3 contains K-feldspar, apatite and titanite inclusions (Supplementary Figure SF.3C), but another Hadean grain (#11) is free of inclusions. These inclusions are not confined within cracks or fissures and are therefore likely primary^43^ although exceptions occur^47^.

**Table 1:** Selected SHRIMP U-Pb and Lu-Hf isotopic data for samples RM-1 and 5 (OMTG).

Sample	^207^Pb/^206^Pb age (Ma)	Discordance (%)	Th/U	^176^Hf/^177^Hf (t)	±error (2 σ)	εHf[t]	±error (2 σ)
RM-1-3-1	4031 ± 5	0	0.44	0.28005	0.00007	−4.1	1.3
RM-1-3-2	4036 ± 15	1	0.55	0.28001	0.00007	−5.2	1.3
RM-1-3-3	4010 ± 6	12	0.44	—	—	—	—
RM-1-3-4	4057 ± 8	4	0.62	—	—	—	—
RM-1-5-1	3368 ± 12	11	0.44	0.28055	0.00007	−1.8	1.3
RM-1-7-1	3364 ± 7	4	1.29	0.28049	0.00007	−4.3	1.3
RM-1-14-1	3402 ± 8	−1	1.10	0.28055	0.00008	−1.3	1.4
RM-1-15-1	3330 ± 23	8	1.78	0.28053	0.00008	−3.6	1.3
RM-1-19-1	3362 ± 10	8	1.98	0.28047	0.00008	−4.9	1.5
RM-1-21-1	3365 ± 8	12	0.47	0.28059	0.00007	−0.8	1.3
RM-1-22-1	3385 ± 6	2	1.45	0.28052	0.00007	−2.8	1.3
RM-1-23-1	3400 ± 10	2	2.03	0.28049	0.00008	−3.2	1.4
RM-5-1-1	3396 ± 8	−3	1.84	0.28057	0.00007	−0.4	1.2
RM-5-2-1	3670 ± 7	5	0.51	0.28022	0.00006	−6.6	1.1
RM-5-3-1	3363 ± 11	0	1.38	0.28054	0.00007	−2.5	1.3
RM-5-5-1	3341 ± 5	10	0.76	0.28053	0.00008	−3.4	1.5
RM-5-6-1	3415 ± 7	−1	0.26	0.28047	0.00010	−3.7	1.8
RM-5-8-1	3386 ± 8	15	1.00	0.28054	0.00007	−1.9	1.3
RM-5-9-1	3673 ± 7	0	0.57	0.28027	0.00008	−4.7	1.4
RM-5-10-1	3396 ± 6	−1	1.02	0.28053	0.00008	−2.0	1.4
RM-5-11-1	4241 ± 4	−1	0.65	0.27995	0.00009	−2.5	1.6
RM-5-11-3	4239 ± 4	0	0.65	—	—	—	—
RM-5-12-1	3390 ± 11	−1	1.60	0.28056	0.00007	−1.0	1.2
RM-5-14-1	3433 ± 6	−1	0.97	0.28051	0.00008	−1.9	1.4
RM-5-15-1	3394 ± 8	0	1.56	0.28054	0.00006	−1.8	1.0
RM-5-16-1	3381 ± 14	−1	1.52	0.28054	0.00006	−1.9	1.1
RM-5-18-1	3394 ± 7	2	1.13	0.28051	0.00007	−2.8	1.3
RM-5-19-1	3595 ± 12	5	0.69	0.28041	0.00007	−1.5	1.3

*^176^Hf/^177^Hf (t) denote initial isotopic ratios calculated using ^207^Pb/^206^Pb ages (Ma) of respective spots in zircons. ^207^Pb/^206^Pb age data (Ma) with ≤10% discordance are presented (including 4 spots with >10% discordance but consistent ^176^Hf/^177^Hf (t) values), whereas all data are presented in Supplementary dataset SF1 and 2.

**Figure 3:**
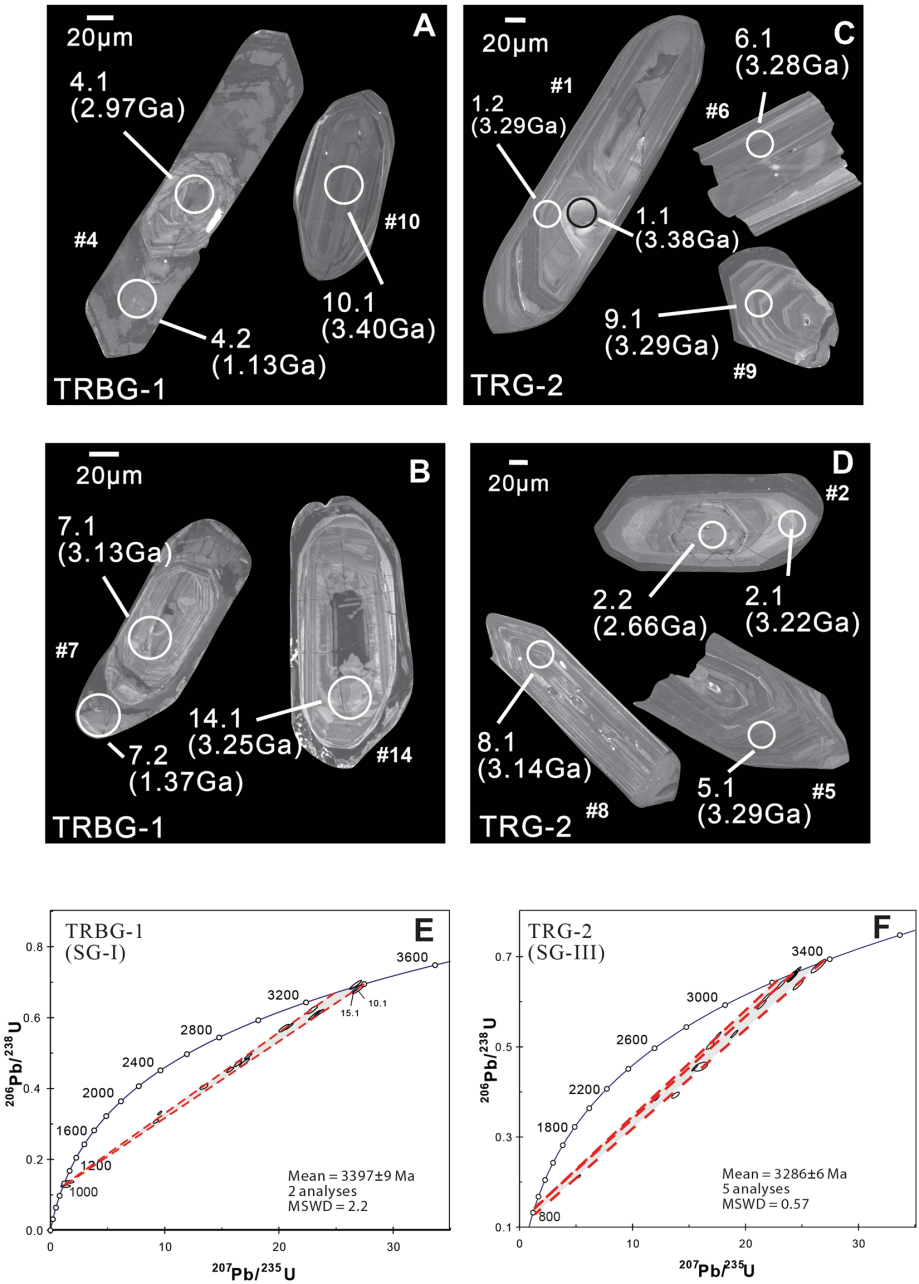
(**A**,**B**) Cathodoluminiscence (CL) image of zircons from Singhbhum granite sample TRBG-1 (SG-I), (**C**,**D**) from sample TRG-2 (SG-III). (**E**,**F**) U-Pb Concordia plots for the zircons from TRBG-1 and TRG-2 respectively (ages represent weighted mean of ages in Ma). Error ellipses are shown at 1σ.

Zircons from the granite gneiss (TRBG-1) of the Singhbhum Granite Phase-I (SBG-I) and another granite (TRG-2), collected from the Singhbhum Granite Phase-III (SBG-III), identified from the regional geological map after Saha^26^ (Fig. 1) are euhedral in shape and mostly exhibit well-developed oscillatory zoned cores with homogeneous rims that are visible in CL images (Fig. 3A–D). Zircons from TRBG-1 yield a ^207^Pb/^206^Pb upper intercept age of 3397 ± 9 Ma (MSWD = 2.2; n = 2; Fig. 3E), where two slightly younger concordant ages (≤10% concordance) of 3267 ± 6 Ma and 3289 ± 10 Ma are also recorded (Table 2). Zircons from TRG-2 record a U-Pb upper intercept age of 3286 ± 6 Ma (MSWD = 0.57; n = 5; Fig. 3F). Zircon grains of sample TRG-2 contain older cores with concordant ages of 3377 ± 11 Ma (grain #1; Fig. 3C) and 3367 ± 7 Ma (grain #10), which are contemporaneous with those of the RM and TRBG samples (Tables 1 and 2). The 3377 ± 11 Ma core in grain #1 (spot 1.1) from TRG-2 is identified as a xenocryst, as it contains markedly lower U concentrations than its rim^43^ (Supplementary Table S1), and the core appears to be much brighter than the rest of the grain in the CL image (Fig. 3C).

**Table 2:** Selected SHRIMP U-Pb and Lu-Hf isotopic data for samples TRBG-1 (SG-I) and TRG-2 (SG-III).

Sample	^207^Pb/^206^Pb age (Ma)	Discordance (%)	Th/U	^176^Hf/^177^Hf (t)	± error (2 σ)	εHf[t]	± error (2 σ)
TRBG-1-1-1	3352 ± 6	8	0.76	0.28060	0.00007	−0.7	1.3
TRBG-1-3-1	3361 ± 7	−1	0.71	0.28068	0.00008	2.6	1.4
TRBG-1-6-1	3248 ± 12	11	0.29	0.28060	0.00008	−3.1	1.4
TRBG-1-8-1	3267 ± 6	10	0.66	0.28061	0.00007	−2.0	1.3
TRBG-1-9-1	3340 ± 6	8	0.63	0.28051	0.00007	−4.0	1.2
TRBG-1-10-1	3404 ± 7	2	0.67	0.28045	0.00007	−4.7	1.3
TRBG-1-13-1	3289 ± 10	5	1.13	0.28056	0.00007	−3.6	1.3
TRG-2-1-1	3377 ± 11	1	0.25	0.28058	0.00006	−0.6	1.1
TRG-2-3-1	3246 ± 6	6	0.77	0.28063	0.00006	−2.1	1.2
TRG-2-4-1	3287 ± 6	0	0.61	0.28060	0.00006	−2.2	1.1
TRG-2-5-1	3285 ± 6	0	0.68	0.28061	0.00006	−1.7	1.1
TRG-2-6-1	3278 ± 7	1	0.99	0.28060	0.00006	−2.5	1.1
TRG-2-7-1	3258 ± 7	8	0.55	0.28061	0.00007	−2.6	1.2
TRG-2-9-1	3291 ± 7	1	0.58	0.28060	0.00009	−1.9	1.6
TRG-2-10-1	3367 ± 7	6	0.63	0.28060	0.00007	−0.3	1.3
TRG-2-14-1	3288 ± 7	1	0.55	0.28057	0.00007	−3.1	1.2
TRG-2-16-1	3264 ± 7	3	0.7	0.28060	0.00007	−2.7	1.2

^*207^Pb/^206^Pb age data (Ma) with ≤10% discordance are presented (including 1 spot with >10% discordance but consistent ^176^Hf/^177^Hf (t)value), whereas all data are presented in Supplementary dataset SF1 and 2.

### Hf isotopic compositions of OMTG and SG zircons

The ^176^Hf/^177^Hf compositions of Hadean xenocrysts (>4.0 Ga) and their host Paleoarchean zircons are summarized in Table 1 and presented in full in Supplementary Table S2. The Hf isotopic analysis of one spot obtained from the oldest Hadean xenocryst (4241 ± 7 Ma; spot 5-11-1) yields a subchondritic^48^ ɛHf[t] value of −2.5 ± 1.6 and is similar to the other two younger Hadean xenocrysts of 4031 ± 5 Ma and 4036 ± 15 Ma, which yield εHf[t] values of −4.1 ± 1.3 and −5.2 ± 1.3, respectively (Table 1). The rim of the ~4.2 Ga xenocryst (grain #11) with a discordant^207^Pb/^206^Pb age of ~3.86 Ga yields an unusual εHf[t] value of −11.9, which probably due to the underestimated age assignment, considering its discordance. However, the initial ^176^Hf/^177^Hf value of this rim (0.27994 ± 0.00008) is remarkably close to that of the Hadean core (0.27995 ± 0.00009) (Supplementary Table S2) indicating same source. Hence, calculating the εHf[t] value of this spot with its upper intercept age of 4.24 Ga as a proxy, yields a value of the ~3.86 Ga rim that is more consistent (−3.2 ± 1.5) with those of the other Hadean zircons. The initial ^176^Hf/^177^Hf value of the oldest Hadean xenocryst (4241 ± 4 Ma) in the OMTG is the least radiogenic (0.27995 ± 0.00009; Table 1). Two younger xenocrysts with ages of 4031 ± 5 and 4036 ± 15 Ma yield slightly more radiogenic, but altogether subchondritic, initial ^176^Hf/^177^Hf values of 0.28005 ± 0.00007 and 0.28001 ± 0.00007, respectively, which are identical within error.

**Figure 4:**
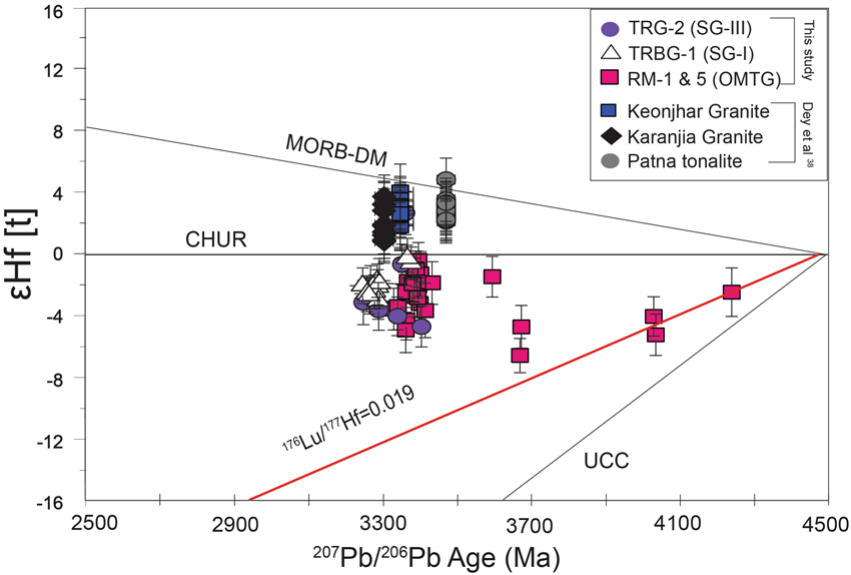
ɛHf[t] vs. ^207^Pb/^206^Pb age (Ma) plot of zircons from samples RM-1, RM-5, TRBG-1 and TRG-2. Data from contemporary Singhbhum TTGs (∼3.5-3.3 Ga) are from Dey *et al*.^39^. Note all data from present study are subchondritic and Hadean and Eoarchean xenocrysts follow ^176^Lu/^177^Hf = −0.019 array. The isotope trajectory of UCC (Upper continental Crust; ^176^Lu/^177^Hf = 0.008) is after Rudnick and Gao^66^.

On the εHf[t] vs ^207^Pb-^206^Pb age (Ma) diagram (Fig. 4), the pre-4 Ga xenocrysts of the OMTG follow an array with a slope of 0.0103, corresponding to a source ^176^Lu/^177^Hf ratio of 0.019 (calculated after Amelin *et al*.^19^), which intersects the chondritic uniform reservoir (CHUR) line at 4.497 ± 0.19 Ga (Fig. 4). The source Lu/Hf ratio calculated from the Hadean zircons, although slightly lower, is consistent with the source being typical mafic crust; that ranges from 0.22^19^ to 0.20^21^ and is far higher than that of the average TTG crust (0.01) calculated from the oldest Jack Hill zircons^20^. The intersection age (4.497 ± 0.19 Ga) of this array with the CHUR reference line is closer to the CHUR extraction age of 4.46 ± 0.12 Ga as the source reservoir of the Jack Hill zircons^21^.

The Eoarchean domains in sample RM-5 with concordant ages of 3673 ± 7 Ma and 3670 ± 7 Ma, yield εHf[t] values of −4.7 ± 1.4 and −6.6 ± 1.1, respectively. Their initial Hf compositions at 3.67 Ga are near identical, e.g., 0.28027 ± 0.00008 and 0.28022 ± 0.00006 respectively, and they are notably higher than those of the Hadean xenocrysts (0.279947–0.280045). However, the oldest Paleoarchean xenocryst, which has a concordant age of 3595 ± 12 Ma, yields an εHf[t] value that is closer to a chondritic value (−1.5 ± 1.3) and a ^176^Hf/^177^Hf_initial_ value (0.28041 ± 0.00007) that is higher than those of the older Hadean and Eoarchean age spots. The ~3.3-3.4 Ga age group of zircons from samples RM-1 and RM-5 yields more radiogenic εHf[t] values than Hadean and Eoarchean age spots, ranging from −0.4 ± 1.2 to −3.7 ± 1.8, except for two data points that fall below −4 epsilon units (−4.9 ± 1.5 and −4.3 ± 1.3). Initial Hf ratios of these spots are identical to those of other age spots with lower εHf[t] values, implying that they were derived from the same source. Initial ^176^Hf/^177^Hf values of the ∼3.3-3.4 Ga zircons display a relatively small range of values, varying between 0.28047–0.28057, identical with the average of 0.28053 ± 0.00003. This range also includes ^176^Hf/^177^Hf ^initial^ values of four discordant age spots (10–15% discordance; Table 1), indicating that despite having undergone U-Pb resetting, their Hf isotopic ratios remain unchanged. The ~3.3-3.4 Ga zircons do not exhibit any particular trend in εHf[t]-time space, but they cluster within the field delimited by the ^176^Lu/^177^Hf = 0.019 array defined by the Hadean zircon data and the CHUR reference line (εHf = 0; Fig. 4). This indicates, unlike Zack Hill zircons, the younger zircons of OMTG are not derived from the same source as the oldest crust.

The ɛHf[t] values of the 3397 ± 9 Ma zircons from sample TRBG-I (SG-I) and 3286 ± 6 Ma zircons from sample TRG-2 (SG-III) are all subchondritic, yielding ɛHf[t] values ranging from −0.7 ± 1.3 to −4.7 ± 1.3 and from −0.3 ± 1.1 to −3.1 ± 1.2, respectively (Table 2), similar to the 3.3-3.4 Ga age group of the OMTG zircons. However, a TRBG-1 analytical site with an age of 3361 ± 7 Ga yields an ɛHf[t] value of + 2.6 ± 1.4 (spot 3.1), which is the only superchondritic value among our entire dataset. The oldest age spot (3404 ± 7 Ma) of the TRBG-1 zircons yields an initial Hf ratio of 0.28045 ± 0.00007 and an ɛHf[t] value of −4.7 ± 1.3. Likewise, the site with an age of 3361 ± 7 Ma yields the highest radiogenic initial Hf composition of 0.28068 ± 0.00008 among the entire zircon population. Apart from these, 6 spots (five with <10% and one with 11% discordance) with ages ranging from 3248 to 3352 Ma yield ^176^Hf/^177^Hf^initial^ values that overlap (within uncertainty) the average value (0.28057 ± 0.00008; Table 1). The initial ^176^Hf/^177^Hf ratios of the sites of the TRG zircons with ages of 3377 to 3246 Ma are identical to their average value of 0.28060 ± 0.00003, within analytical error (Table 2). The average initial ^176^Hf/^177^Hf ratios of TRBG-1 (0.28057 ± 0.00008) and TRG-2 (0.28060 ± 0.00003) are also comparable Table-1.

## Discussion

Interestingly, the subchondritic Hf composition (ɛHf[t] < 0) of the oldest (4241 ± 4 Ma) xenocryst indicates the presence of a non-chondritic mantle reservoir as early as ∼4.2 Ga. The composition of the earliest mantle reservoirs of Earth has remained controversial. The reported initial ^176^Hf/^177^Hf ratios of the Bulk Silicate Earth (BSE), i.e., 0.279685 ± 19^49^ or 0.279781 ± 18^22^, which are lower than that of the chondritic reservoir, argue against the decades-old paradigm of the chondritic Earth and are explained by the accelerated decay of ^176^Lu^50,51^. However, such accelerated decay is caused by the high rate of irradiation of chondritic or eucritic meteorites by γ and/or galactic cosmic rays, a process whose effectiveness has been questioned for the BSE^52^ due to the restricted penetration depth of these rays^51,53^. Alternatively, it has been assumed that the Earth was developed from chondritic material but was subsequently modified by either collisional erosion during accretion^54,55^ or explosive basaltic volcanism in planetesimals^56^. Hence, we assume that the source reservoir of the Hadean OMTG xenocrysts was initially separated from chondritic material, and we interpret our zircon data considering CHUR^48^ as a reference frame. The source array of Lu/Hf = 0.019 fitted through these xenocrysts is comparable to ‘mafic protocrust’ with ^176^Lu/^177^Hf values proposed by Kemp *et al*.^21^ (0.020) and Amelin *et al*.^19^ (0.022) calculated from Jack Hill detrital zircon data. The age of separation (∼4.5 Ga) of the enriched reservoir from the chondritic reservoir calculated from the Hadean zircons in this study is also very similar to the CHUR separation age of the source reservoir of the Jack Hill zircons (∼4.5 Ga^24^ or 4.46 ± 0.12 Ga^21^). The development of the ~4.49 Ga enriched reservoir recorded in the Singhbhum craton is also in near-agreement with the estimated age of ~4.5 Ga for the separation of the enriched silicate reservoir upon Earth’s solidification, based on recent geodynamic modeling^57–59^.

Assuming that the parental magma of these zircons is likely to be felsic due to the high solubility of zirconium in mafic-ultramafic magmas^60^, the Hadean (~4.2-4 Ga) zircons of the OMTG were presumably generated from minor silicic melts produced as a consequence of the differentiation or re-melting of pre-4.2 Ga juvenile protocrust of mafic composition. The formation and reworking of juvenile crust were either contemporaneous or separated by a short period of ~100-300 My during the Hadean and Archean eons^49^. To explain the nature of the enriched mantle reservoir that parented the Hadean OMTG zircons, we envisage that such a reservoir may represent an enriched, residual mafic magma generated from a partially solidified magma ocean, analogous to KREEP beneath the lunar anorthositic crust^20,21,61–63^. This mafic protocrust was presumably reworked and re-melted to generate felsic melt between ~4.2-4.0 Ga without the significant addition of juvenile material from the mantle. The mineral inclusions in the Hadean OMTG xenocrysts, including K-feldspar, titanite and apatite (Supplementary Figure SF3C), were likely generated from a differentiated melt. The existence of Hadean mafic protocrust has previously been estimated based on Hadean to Paleoarchean Jack Hill’s zircons^19–21^, ~3.7 Ga metasediments from Isua^64^ and detrital zircons from the Pilbara^65^. Similarly, the source ^176^Lu/^177^Hf (0.019) calculated from the OMTG validates that a mafic-dominated crust prevailed in the Singhbhum Craton during the Hadean, negating the possibility of the significant presence of upper continental (^176^Lu/^177^Hf = 0.008^66^) or ‘TTG-like’ crust (^176^Lu/^177^Hf = 0.01^20^).

Thus, it is necessary to determine the fate of this ancient, enriched Hadean reservoir in the Singhbhum Craton. The Eoarchean (~3.6 Ga) zircon age domains in sample RM-5 record the second-oldest stage of felsic melt generation; these are slightly more radiogenic than the Hadean ones. Therefore, they were probably generated from the modification of the enriched source of Hadean zircons due to its interactions with juvenile (more radiogenic) mantle melt, as is evidenced by the fact that the ɛHf[t] and ^176^Hf/^177^Hf^initial^ compositions of these sites (Table 1) are higher than those of the Hadean ones. This also invokes the assumption that the composition of the enriched, subchondritic mantle reservoir in the Singhbhum Craton persisted without undergoing modification until the Eoarchean (~3.7 Ga). However, the identifiable vertical excursion of εHf[t] values in the time-integrated εHf[t] plot (Fig. 4) in the Paleoarchean (3.3-3.4 Ga) for OMTG zircons and other contemporary Singhbhum TTGs, such as TRBG-1 (~3.4 Ga) and TRG-2 (~3.3 Ga), to near-chondritic values confirms the variable mixing of mantle-derived, juvenile material with material from the old, enriched reservoir during the period between 3.4 and 3.3 Ga. However, the average ^176^Hf/^177^Hf^initial^ values of the 3330-3433 Ma zircons in samples RM-1 & RM-5 (OMTG), the 3267-3352 Ma zircons in samples TRBG (SG-I) and the 3377-3246 Ma zircons in samples TRG-2 (SG-III) are 0.28053 ± 0.00006, 0.28057 ± 0.00008 and 0.28060 ± 0.00003, respectively. These values are closely comparable except for one spot with an age of 3404 Ma with a slightly less radiogenic ^176^Hf/^177^Hf^initial^ ratio of 0.28041. Clearly, these Paleoarchean zircons were derived from felsic melts with near identical Hf isotopic values, while minor disparity is most likely due to incomplete mixing between the enriched reservoir with depleted juvenile magma. The results of a previous petrological modeling study^67^ suggested that the protolith of the OMTG was generated by the 40% partial melting of the OMG amphibolites at garnet stability depths^32^. It is unlikely that the the remnants of the earliest Hadean mafic protocrust survived the constant reworking processes until today. Remnants of the oldest mafic protocrust may have been preserved in the amphibolite enclaves within the OMTG or these enclaves could represent a modified mafic component developed from interactions between the ancient enriched reservoir and mantle-derived, juvenile mafic magma and was preserved as melting residuum of the mafic protolith from which OMTG magma was generated. Interestingly, the zircons from the ~3.5 to ~3.3 Ga TTGs of the Singhbhum Craton, which are located near Keonjhar, exhibit suprachondritic Hf isotopic signatures with average ɛHf[t] values ranging from +2.9 to +2.2^39^, suggesting that they were derived from a depleted source reservoir. However, this implies that a separate depleted reservoir, which was probably complementary with the ancient, enriched reservoir hypothesized in the present study, of Paleoarchean (~3.5 Ga) or even older age, existed under the cratonic lithosphere of the Singhbhum craton and also participated in the generation of TTG magma.

Based on isotopic constraints, it has been suggested that Earth’s accretion was roughly complete 30 Myr after^68,69^ the condensation of the oldest solids from the solar nebula at ${4568.2}_{-0.4}^{+0.2}$ Ma^70^. Hence, the separation of the enriched reservoir (4.497 ± 0.19 Ga) in the Singhbhum Craton occurred soon after (~40 My) the accretion of the Earth. Evidence from short-lived isotopes, e.g., ^146^Sm-^142^Nd^71–74^ or ^182^Hf-^182^W^75,76^, and long-lived isotopic systems (^176^Lu-^176^Hf^21,77,78^) suggest that the development of enriched and depleted reservoirs occurred very early in Earth’s history, probably within 100–200 Ma of planetary accretion, and that complementary enriched (Early Enriched Reservoir, or EER) and depleted reservoirs (Early Depleted Reservoir, or EDR) existed during the Hadean^64,71–79^. The nature and fate of this EER remains elusive, although its presence has been deduced from the ~3.4 Ga Ameralik dykes of the Amitsoq complex^80^. The highly unradiogenic, Hadean zircons of the OMTG most likely represent product of the EER that existed in the Singhbhum cratonic mantle at ~4.2-4 Ga. The presence of ∼3.3-3.4 Ga TTG zircons (OMTG and SG) with unradiogenic Hf signals in this study indicates that this enriched reservoir was sustained until the Paleoarchean. Therefore, the development of the EER from the chondritic mantle at ∼4.5 Ga raises possible questions about the existence of the complementary depleted mantle. Evidence of depleted mantle under the Singhbhum Craton during the Paleoarchean has already been recorded in ∼3.5-3.4 Ga zircon with radiogenic Hf isotope signatures from the TTGs of the Singhbhum Craton^39^.

Is it possible to back-track the oldest depleted Hadean reservoir that is complementary to the enriched one recorded in the Hadean zircons in this study? The most plausible candidate might be the Paleoarchean komatiites (~3.4 Ga) of the Eastern IOG belt, which are the most direct representatives of the Paleoarchean depleted lower mantle (εNd[t] +2 to +4) below the Singhbhum Craton^81^. These komatiites may have separated from much older Hadean mantle and may have recorded evidence of early silicate differentiation, i.e., by preserving a record of the Hadean EDR in the highly radiogenic (εHf[t] = up to +8.2) Paleoarchean (~3.5 Ga) Pilbara komatiites^82^.

U-Pb age constraints clearly indicate that the large-scale generation of continental crust of TTG composition started at ∼3.4-3.3 Ga in the Singhbhum Craton^37–39^. Felsic rocks generated before this time have probably now been completely reworked and recycled, as zircons older than this are only found as xenocrysts here and in earlier studies^37^. A xenocryst with an age of ∼3.38 Ga is found (grain #1, spot 1.1; Fig. 3C) to be surrounded by an oscillatory zoned zircon rim with an age of ∼3.3 Ga (grain #1; spot 1.2) in sample TRG-2. The site where sample TRG-2 was collected exhibits ubiquitous enclaves of material similar to TRBG-1 (Supplementary Figure SF3C), which suggests that this ∼3.38 Ga xenocryst may have been inherited from SG-I, as it was reworked during the emplacement of the younger SG-III. The oldest concordant age of sample TRG-2 (∼3.29 Ga; grain #20, 23) is equivalent to the 3289 ± 10 Ma age spot (spot 13.1), which indicates that probably this age of the tectonomagmatic event that led to reworking of pre-existing SG-I and emplacement of SG-III.

Before the emergence of dominantly TTG crust, mafic protocrust likely prevailed as a thin, buoyant tectonic plate^83^. It is still unclear whether such proto-plates were stagnant, as the heat production of the Earth’s mantle was more than three times greater during the Archean^84^, which led to more rapid mantle convection, thus triggering faster plate movement. Rapid plate movement invokes the possibility of the quick recycling of thin Hadean protocrust, thus preventing its preservation^85^. Hence, it is possible that the Hadean mafic protocrust in the Singhbhum craton may not have survived long and was recycled and assimilated into more voluminous TTG magma that was generated from a combined process involving the reworking of older, enriched crust and the serial addition of mantle-derived melt during ~3.4-3.3 Ga. The tectonic processes involved in the partial melting of the OMG amphibolites to generate the parent magma of the OMTG are still unclear. However, the geochemical data of the ~3.5-3.3 Ga TTGs of the Singhbhum craton suggest that these TTGs lack the signatures of subduction-derived magma; they are thus considered to have been generated from the reworking of pre-existing mafic crust by the repeated underplating of plume-derived mafic-ultramafic magma^39^ during the Paleoarchean^81^.

## Conclusions

The combined U-Pb SHRIMP and Lu-Hf isotopic data of the ~4.24 and ~4.03 Ga xenocrystic zircons from the ~3.4 Ga TTG of the ‘Older Metamorphic Tonalitic Gneiss (OMTG)’ of the Archean Singhbhum Craton of Eastern India contain records of the oldest crust in India. The essentially subchondritic (ɛHf[t] < 0) isotopic signatures of these Hadean zircons indicate that they originated from the reworking of older crust prior to ~4.2 Ga. The calculated ^176^Lu/^177^Hf ratio (0.019) of their source reservoir indicates the mafic nature of the older crust that originated from an enriched reservoir that separated from the chondritic reservoir at ~4.5 Ga. However, the younger and almost contemporaneous zircons from the OMTG (3.3-3.4 Ga), Singhbhum Granite Phase-I (SG-I; ~3.4 Ga) and Singhbhum Granite-III (SG-III; ~3.3 Ga) yield more radiogenic Hf isotopic signatures, indicating that this enriched reservoir persisted but underwent mixing with juvenile mantle material during the Eo-Paleoarchean.

## Methods

Zircon separation, CL imaging, inclusion analysis and SHRIMP U-Pb dating were carried out at the Beijing SHRIMP Center, Institute of Geology, Chinese Academy of Geological Sciences, using a SHRIMP II following the analytical procedures described by Williams^86^. Zircon crystals were obtained using standard crushing and grinding techniques, followed by separation using heavy liquid and magnetic techniques. The hand-picked crystals were cast in epoxy resin discs and polished. The intensity of the primary O^2−^ ion beam was 5 nA and the spot size was 25–30 μm; each site was rastered for 150 s prior to analysis. Five scans through the mass stations were made for each age determination. The standard used for the calibration of elemental abundances was M257, which contains U = 840 ppm^87^. TEMORA, whose ^206^Pb/^238^U age is 417 Ma^88^, was analyzed for the calibration of ^206^Pb/^238^U ratios after every 3 analyses. Detailed CL images of these zircons were captured. All grains were imaged using a CARL-ZEISS MERLIN Compact with GATAN Mono CL4, and the inclusions within Hadean zircons were analyzed using the same scanning electron microscope with OXFORD IE250. The data were processed and assessed using the Squid 1.02^89^ and Isoplot 3.00^90^ programs. Common Pb corrections were based on the measured ^204^Pb contents. The errors given in Table 1 and the concordia intercept ages for individual analyses are quoted at the 1σ level, whereas the errors for weighted mean ages in the text are quoted at the 95% confidence level.

The in situ Lu-Hf analyses of zircons from all four TTG samples were conducted on the pits generated during U-Pb dating at the State Key Laboratory of Lithospheric Evolution, Institute of Geology and Geophysics, Chinese Academy of Sciences, Beijing, using a 193 nm UV ArF excimer laser ablation system attached to a Neptune multi-collector ICP MS. The instrumental conditions and analytical procedures were described by Wu *et al*.^91^. Each measurement included an ablation time of ∼26 s for 200 cycles, a repetition rate of 6–8 Hz, a laser power of 100 mJ/pulse and a spot size with a diameter of 44 μm. Helium was used as the carrier gas for the ablated aerosols. The average ^176^Hf/^177^Hf ratios of the Mud Tank^92^ and Plešovice^93^ standards obtained in this study after repetitive analyses were 0.282500 (n = 25) and 0.282484 (n = 18), respectively. All Lu–Hf isotopic results are reported with 95% confidence limits.

The calculation of εHf[t] values was based on the ^207^Pb/^206^Pb SHRIMP spot analysis ages, chondritic values (^176^Hf/^177^Hf = 0.282785, ^176^Lu/^177^Hf = 0.0336^48^) and a ^176^Lu decay constant of 1.865 × 10^−11^ year^−1^ ^94^. Selected ^207^Pb/^206^Pb concordant and some discordant (10–15% discordant) age data of zircon spots with Hf isotope values consistent with concordant ones are summarized in Tables 1 and 2. All U-Pb age data and Lu-Hf isotopic data are listed in the Supplementary Material SF1 and 2. During interpretation, zircon ^207^Pb/^206^Pb age data with >10% U-Pb discordance and Th/U ratios of <0.15 were commonly disregarded. However, some discordant data, such as those with ^176^Hf/^177^Hf ratios identical to those of the concordant population, were included because although their U-Pb ratios have been modified, their original Lu-Hf isotopic ratios were preserved.

### Availability of materials and data

All data generated or analysed during this study are included in this published article and its Supplementary Tables (SF1 and 2).

## Electronic supplementary material

Supplementary Information
