# Supplementary material for: Evidence of Enriched, Hadean Mantle Reservoir from 4.2-4.0 Ga zircon xenocrysts from Paleoarchean TTGs of the Singhbhum Craton, Eastern India
Source: Sci Rep. 2018 May 4;8:7069. doi: 10.1038/s41598-018-25494-6 (PMC5935743; doi:10.1038/s41598-018-25494-6)
Supplement: Supplementary file 1 — Supplementary Information [file 41598_2018_25494_MOESM1_ESM.pdf]

Supplementary information

Evidence of Enriched, Hadean Mantle Reservoir from 4.2-4.0 Ga old zircon xenocrysts from Paleoarchean TTGs of the Singhbhum Craton, Eastern India.

Trisrota Chaudhuri[1], Yusheng Wan[2], Rajat Mazumder[3, *], Mingzhu Ma[2] and Dunyi Liu[2]

1.Deartment of Geology, University of Calcutta, 35 Ballygunge Circular Road, Kolkata, India.
2.Beijing SHRIMP Centre, Institute of Geology, Chinese Academy of Geological Sciences, Beijing, 100037, China
3.Department of Applied Geology, Faculty of Engineering and Science, Curtin University of Technology Sarawak, CDT 250, Miri 98009, Sarawak, Malaysia.

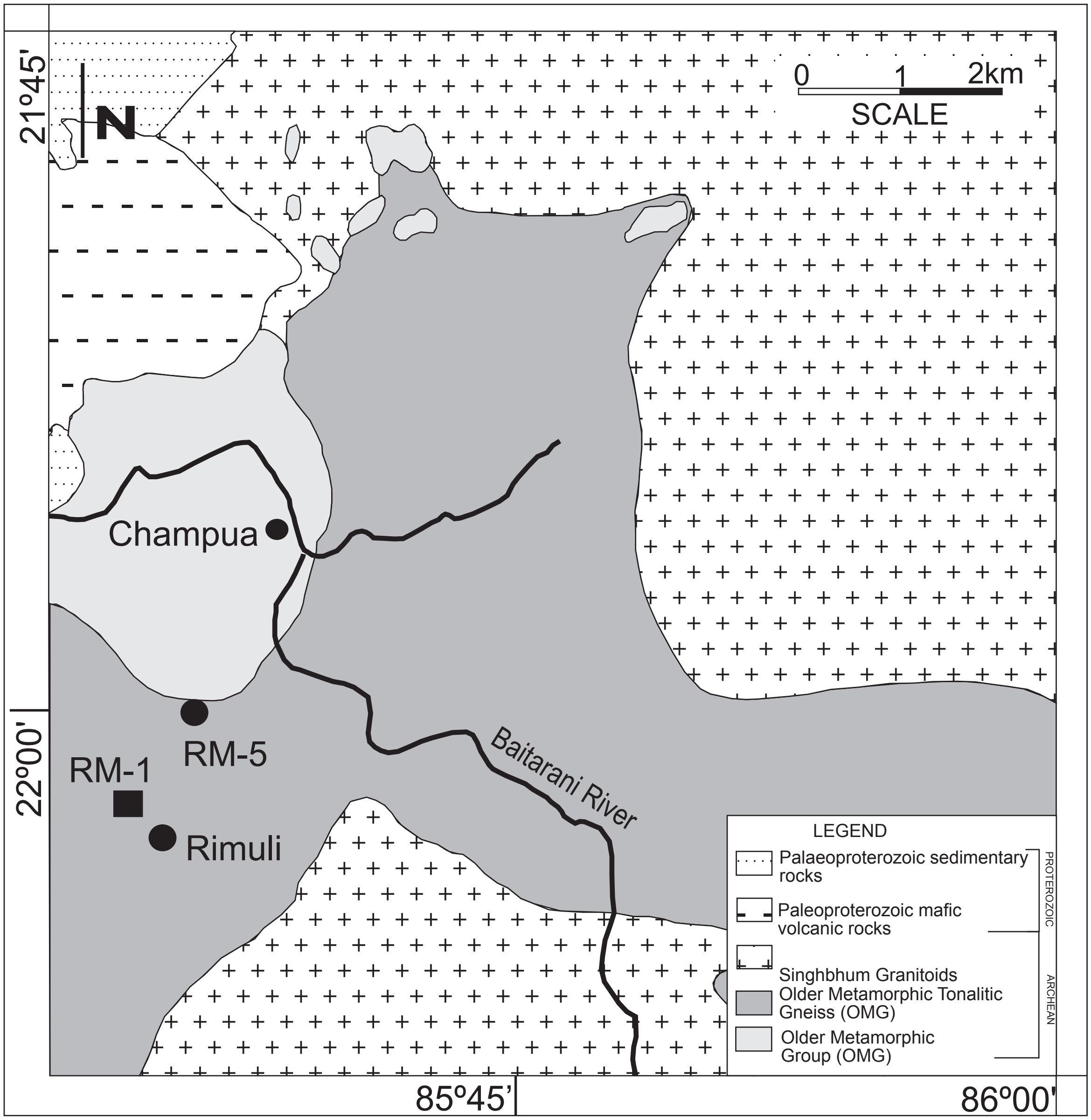

Supplementary Figure SF1: Geological map of Rimuli-Champua area showing locations of Older Metamorphic Tonalitic Gneiss samples RM-1 and 5 containing Hadean zircon xenocrysts. The map was created by the author Trisrota Chaudhuri during her fieldwork and was modified using software Corel Draw® Graphics Suite X7.

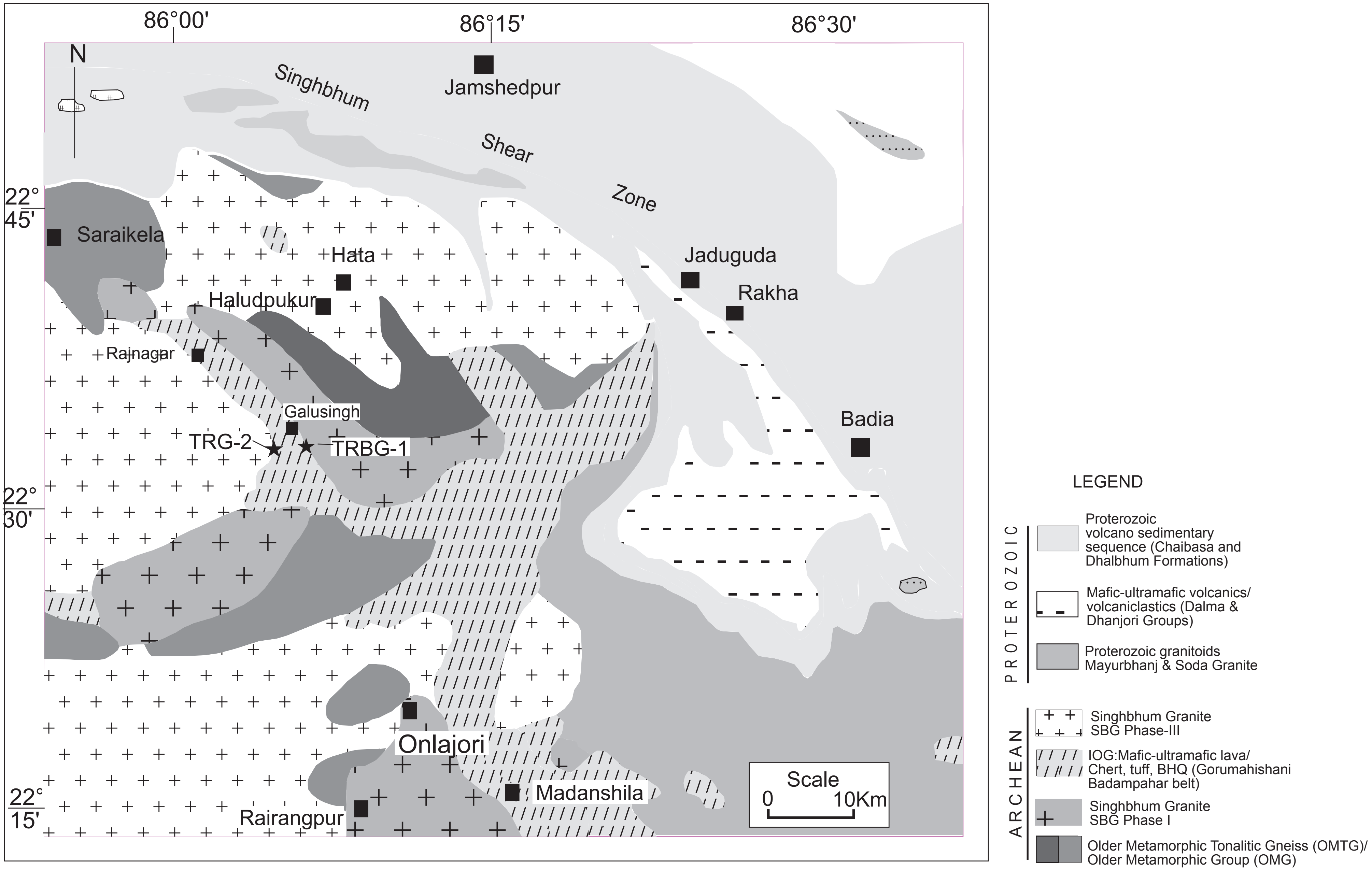

Supplementary Figure SF2: Geological map of eastern part of Singhbhum Craton showing locations of Paleoarchean samples TRBG-1 (Singhbhum Granite-I) and TRG-2 (Singhbhum Granite-III) (modified after Upadhyay et al.[37] using Corel Draw Graphic Suite X7).

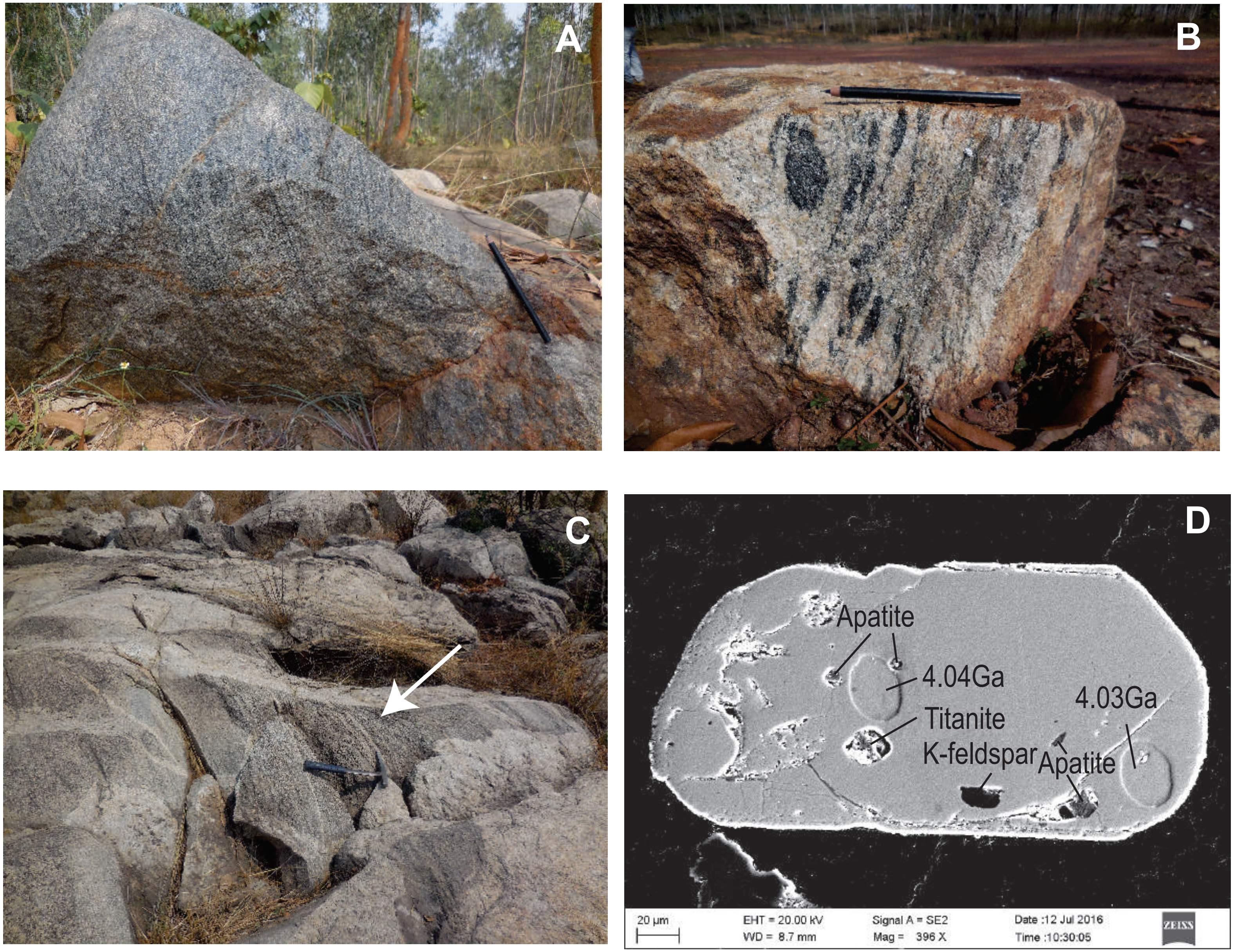

Supplementary Figure SF3: A.) Field photograph of site of sample RM-1, B.) Amphibolite enclaves within OMTG near location of RM-5, C.) Field photograph of enclave of sample TRBG-1 (white arrow) within TRG-2, D) Inclusions within ~4.03 Ga old zircon xenocryst (Sample-RM-1, grain #3).

Supplementary file SF4: Petrographic description of samples RM-1, 5, TRBG-1 and TRG-2.

RM-1 & 5

In these rocks, grains are partially recrystallized and primary igneous texture and grain shapes are partially modified by deformation and grain boundaries are sutured. Average grain size is medium (1-5 mm). On average, samples RM-1 and 5 consist of ~ 40 modal% plagioclase, ~33 modal% quartz, ~15modal% K-feldspar, ~3 modal% microcline, ~3 modal% biotite, ~2 modal% muscovite (secondary), ~1 modal% amphibole ~2 modal % pyrite and magnetite and ~1 modal% represented by garnet, apatite, zircon, titanite, allanite and monazite. Plagioclase grains are subhedral, sassuritized or partially replaced by secondary calcite. Quartz is embayed and exhibits undulose extinction/subgrain formation and recrystallization at grain margins. K-feldspar grains are subhedral and are partially kaolinized. Biotite is fine grained (≤1 mm), subhedral, brown to reddish brown in colour, deformed and commonly altered to chlorite. Muscovite grains are fine to medium (<1-2 mm) grained and are also subhedral to nearly euhedral and replace biotite and plagioclase. Small (<1 mm), euhedral magnetite and pyrite grains occur as disseminations within these rocks. Hornblende is medium-grained (1-5 mm) subhedral and rare.

TRBG-1

This rock is a feldspathic augen gneiss with megacrystic (5mm-1cm), plagioclase feldspar augen within a coarse grained matrix. Biotite flakes are aligned along the gneissic foliation. This rock is highly weathered and gneissosity is only discernible at rare fresh exposures .The rock is coarse grained with an inequigranular texture and consists of coarse (5mm-1 cm), subhedral, plagioclase megacrysts set within a matrix of plagioclase (~ 35-40 modal%), quartz (~25-30 modal%), K-feldspar (~22-23 modal%), microcline (~5-10 modal%), biotite (~1 modal%) with accessory titanite, apatite, monazite and zircon (together ~1 modal%) as accessory minerals. Plagioclase in the groundmass is medium- coarse grained (1-5 mm), subhedral to anhedral and, together with plagioclase megacrysts, exhibit deformation twin lamellae and marginal granulation as evidence of deformation. Similarly, quartz grains are also partially recrystallized.

TRG-2

Sample TRG-2 consists of microcline (~35 modal%), plagioclase (~20 modal%), quartz (~30 modal%), K-feldspar (~3 modal%), biotite (~1 modal%), apatite, monazite, zircon (together ~1 modal%) and display medium to coarse grained with a granular texture. Microcline is coarse (~5 mm - <1 cm) grained, subhedral and partially keolinized.

Supplementary Table. S1: SHRIMP U-Pb age data of zircons from samples RM-1, RM-5, TRBG1 and TRG-2 from Singhbhum Craton, Eastern India.

Spot	$^{206}Pb_c$ (%)	U (ppm)	Th (ppm)	Th /U	$^{206}Pb^*$ (ppm)	$^{207}Pb^*/^{206}Pb^*$	±%	$^{207}Pb^*/^{235}U$	±%	$^{206}Pb^*/^{238}U$	±%	err corr	$^{206}Pb/^{238}U$ Age (Ma)		$^{207}Pb/^{206}Pb$ Age (Ma)		Discordance (%)
RM-1-1.1	1.08	369	335	0.94	170	0.27142	0.54	19.81	1.2	0.5290	1.1	0.90	2737	±24	3315	± 8	17
RM-1-2.1	0.50	78	94	1.25	37	0.27814	0.81	20.86	1.7	0.5438	1.4	0.87	2799	±33	3353	± 13	17
RM-1-3.1	0.10	202	85	0.44	151	0.43383	0.32	51.95	1.2	0.8680	1.2	0.96	4030	±35	4031	± 5	0
RM-1-3.2	0.14	59	32	0.55	44	0.43542	1.00	51.55	1.9	0.8590	1.5	0.84	3996	±46	4036	± 15	1
RM-1-3.3	0.02	115	49	0.44	72	0.42800	0.42	43.01	1.1	0.7289	1	0.92	3529	±27	4010	± 6	12
RM-1-3.4	0.02	62	37	0.62	44	0.44152	0.53	50.31	1.5	0.826	1.4	0.93	3883	±40	4057	± 8	4
RM-1-3.5	8.30	691	41	0.06	84	0.11511	8.8	2.00	8.9	0.1269	1.3	0.15	770	± 9	1871	±160	59
RM-1-4.1	3.79	197	193	1.01	85	0.26601	1.10	17.52	1.6	0.4762	1.2	0.77	2510	±25	3288	± 16	24
RM-1-5.1	1.64	211	90	0.44	109	0.28072	0.81	22.84	1.4	0.5896	1.2	0.83	2988	±28	3368	± 12	11
RM-1-6.1	0.53	399	340	0.88	118	0.22121	0.49	10.45	1.3	0.3426	1.2	0.93	1899	±20	2990	± 8	36
RM-1-7.1	0.30	202	253	1.29	113	0.28022	0.47	25.01	1.3	0.6472	1.2	0.93	3217	±30	3364	± 7	4
RM-1-8.1	22.88	823	1059	1.33	181	0.15204	11.00	4.09	11.0	0.1930	2.3	0.21	1137	±24	2388	±180	52
RM-1-9.1	0.93	389	277	0.73	152	0.24773	0.50	15.35	1.2	0.4493	1.1	0.92	2392	±23	3171	± 8	25
RM-1-10.1	0.30	255	310	1.26	122	0.26774	1.10	20.46	2.0	0.5542	1.7	0.83	2843	±38	3293	± 17	14
RM-1-11.1	5.52	529	242	0.47	150	0.21931	1.40	9.40	1.7	0.3098	1.1	0.65	1740	±17	2982	± 21	42
RM-1-12.1	0.51	401	399	1.03	188	0.30101	0.36	22.48	1.1	0.5416	1.1	0.95	2790	±24	3476	± 6	20
RM-1-13.1	4.68	247	209	0.88	82	0.25852	1.40	13.13	1.8	0.3667	1.2	0.67	2014	±21	3244	± 21	38
RM-1-14.1	0.05	113	120	1.10	68	0.28712	0.52	27.99	1.5	0.7070	1.4	0.94	3447	±38	3402	± 8	-1
RM-1-15.1	3.73	225	389	1.78	124	0.27381	1.50	22.96	2.0	0.6076	1.3	0.67	3060	±31	3330	± 23	8
RM-1-16.1	12.72	677	625	0.95	123	0.12900	7.80	3.27	7.9	0.1828	1.4	0.18	1082	±14	2091	±140	48
RM-1-17.1	3.12	445	388	0.90	144	0.23403	1.00	11.70	1.5	0.3616	1.1	0.75	1990	±19	3084	± 16	35
RM-1-18.1	1.64	374	479	1.32	127	0.23790	1.20	12.75	1.6	0.3883	1.1	0.70	2115	±20	3108	± 18	32
RM-1-19.1	0.62	204	392	1.98	109	0.27974	0.63	23.77	1.3	0.6162	1.2	0.88	3095	±29	3362	± 10	8
RM-1-20.1	2.52	278	336	1.25	112	0.26961	1.50	16.86	1.9	0.4524	1.2	0.64	2406	±23	3308	± 22	27
RM-1-21.1	1.10	261	120	0.47	132	0.28021	0.51	22.44	1.2	0.5805	1.1	0.92	2951	±27	3365	± 8	12
RM-1-22.1	0.06	186	261	1.45	108	0.28403	0.40	26.44	1.5	0.6754	1.5	0.96	3327	±38	3385	± 6	2
RM-1-23.1	0.25	89	174	2.03	52	0.28671	0.64	26.88	1.5	0.6801	1.4	0.91	3345	±36	3400	± 10	2

Spot	$^{206}Pb_c$ (%)	U (ppm)	Th (ppm)	Th /U	$^{206}Pb^*$ (ppm)	$^{207}Pb^*$ $/^{206}Pb^*$	±%	$^{207}Pb^*$ $/^{235}U$	±%	$^{206}Pb^*$ $/^{238}U$	±%	err corr	$^{206}Pb/^{238}U$ Age (Ma)		$^{207}Pb/^{206}Pb$ Age (Ma)		Discordance (%)
RM-5-1.1	0.03	89	160	1.84	55	0.28601	0.51	28.26	1.4	0.7167	1.3	0.93	3484	±35	3396	± 8	-3
RM-5-2.1	0.13	117	57	0.51	72	0.34162	0.43	33.80	1.3	0.7177	1.3	0.94	3487	±34	3670	± 7	5
RM-5-3.1	0.29	55	73	1.38	33	0.28011	0.70	26.60	1.7	0.6890	1.5	0.91	3379	±40	3363	±11	0
RM-5-4.1	1.39	380	319	0.87	145	0.24702	0.53	14.85	1.2	0.4356	1.1	0.90	2331	±21	3167	± 8	26
RM-5-5.1	0.19	328	242	0.76	168	0.27603	0.33	22.66	1.1	0.5952	1.1	0.96	3011	±26	3341	± 5	10
RM-5-6.1	0.10	149	38	0.26	90	0.28950	0.42	28.06	1.4	0.7031	1.3	0.95	3432	±34	3415	± 7	-1
RM-5-7.1	2.54	473	629	1.38	159	0.23221	0.73	12.16	1.3	0.3790	1.1	0.84	2072	±19	3071	±11	33
RM-5-8.1	0.93	210	203	1.00	103	0.28392	0.51	22.11	1.3	0.5644	1.1	0.92	2885	±27	3386	± 8	15
RM-5-9.1	0.02	93	52	0.57	61	0.34239	0.48	36.03	1.5	0.7630	1.4	0.95	3656	±40	3673	± 7	0
RM-5-10.1	0.06	199	197	1.02	121	0.28602	0.38	27.87	1.2	0.7067	1.2	0.95	3446	±31	3396	± 6	-1
RM-5-11.1	0.11	160	101	0.65	130	0.49981	0.30	65.17	1.2	0.9460	1.2	0.97	4291	±37	4241	± 4	-1
RM-5-11.2	1.52	348	83	0.25	174	0.38652	0.39	30.16	0.7	0.5659	0.6	0.87	2891	±15	3858	± 5	25
RM-5-11.3	0.02	132	83	0.65	105	0.49921	0.27	63.78	1.0	0.9266	0.9	0.96	4227	±29	4239	± 4	0
RM-5-11.4	2.02	365	142	0.40	163	0.38291	0.47	26.48	0.8	0.5014	0.7	0.85	2620	±14	3844	± 6	32
RM-5-11.5	4.99	292	78	0.28	147	0.40502	0.87	30.28	1.1	0.541	0.8	0.76	2787	±18	3931	±10	29
RM-5-12.1	0.28	60	93	1.60	36	0.28501	0.68	27.42	1.7	0.6980	1.5	0.91	3413	±40	3390	±11	-1
RM-5-13.1	3.36	415	668	1.66	145	0.24341	0.92	13.12	1.4	0.3897	1.2	0.81	2121	±21	3147	±14	33
RM-5-14.1	0.10	234	221	0.97	143	0.29300	0.36	28.60	1.2	0.7081	1.1	0.95	3451	±30	3433	± 6	-1
RM-5-15.1	0.15	100	152	1.56	60	0.28562	0.53	27.35	1.4	0.6947	1.3	0.93	3400	±35	3394	± 8	0
RM-5-16.1	0.24	47	69	1.52	28	0.28331	0.92	27.36	1.9	0.7000	1.7	0.87	3422	±44	3381	±14	-1
RM-5-17.1	5.63	545	348	0.66	181	0.23100	1.70	11.55	2.0	0.3609	1.2	0.60	1987	±20	3066	±25	35
RM-5-18.1	0.15	176	192	1.13	102	0.28572	0.46	26.45	1.3	0.6715	1.2	0.93	3311	±31	3394	± 7	2
RM-5-19.1	1.00	79	53	0.69	49	0.32521	0.76	31.52	1.6	0.7030	1.4	0.89	3432	±38	3595	±12	5

Spot	$^{206}Pb_c$ (%)	U (ppm)	Th (ppm)	Th /U	$^{206}Pb^*$ (ppm)	$^{207}Pb^*$ $/^{206}Pb^*$	±%	$^{207}Pb^*$ $/^{235}U$	±%	$^{206}Pb^*$ $/^{238}U$	±%	err corr	$^{206}Pb/^{238}U$ Age (Ma)		$^{207}Pb/^{206}Pb$ Age (Ma)		Discordance (%)
TRBG-1-1.1	0.09	254	186	0.76	134	0.27812	0.39	23.49	1.2	0.6126	1.1	0.95	3080	±28	3352	± 6	8
TRBG-1-2.1	1.29	426	226	0.55	123	0.20971	0.66	9.55	1.2	0.3301	1.1	0.86	1839	±17	2905	± 10	37
TRBG-1-3.1	0.11	159	110	0.71	95	0.27974	0.46	26.69	1.3	0.6920	1.2	0.94	3390	±32	3361	± 7	-1
TRBG-1-4.1	4.91	510	501	1.01	142	0.21809	1.30	9.19	1.6	0.3048	1.1	0.68	1715	±17	2972	± 19	42
TRBG-1-4.2	22.87	1175	392	0.34	163	0.07704	21.00	1.32	21.0	0.1235	1.8	0.09	751	±13	1128	±420	33
TRBG-1-5.1	0.38	275	281	1.05	116	0.25591	0.49	17.29	1.2	0.4900	1.1	0.92	2571	±24	3222	± 8	20
TRBG-1-6.1	1.72	174	49	0.29	87	0.26001	0.78	20.33	1.5	0.5665	1.2	0.86	2893	±29	3248	± 12	11
TRBG-1-7.1	4.13	375	198	0.55	136	0.24060	1.30	13.35	1.7	0.4009	1.2	0.68	2173	±21	3129	± 20	31
TRBG-1-7.2	24.16	1544	382	0.26	239	0.08804	16.00	1.63	16.0	0.1351	1.9	0.12	817	±15	1374	±300	41
TRBG-1-8.1	0.21	312	200	0.66	154	0.26338	0.38	20.87	1.2	0.5747	1.1	0.94	2927	±26	3267	± 6	10
TRBG-1-9.1	0.08	244	149	0.63	128	0.27602	0.39	23.10	1.5	0.6071	1.5	0.97	3058	±36	3340	± 6	8
TRBG-1-10.1	0.10	167	109	0.67	98	0.28752	0.43	26.93	1.3	0.6794	1.2	0.94	3342	±31	3404	± 7	2
TRBG-1-11.1	1.85	334	198	0.61	132	0.25210	0.65	15.68	1.3	0.4505	1.1	0.87	2397	±22	3200	± 10	25
TRBG-1-12.1	1.45	310	145	0.48	127	0.25328	0.66	16.36	1.3	0.4680	1.1	0.87	2475	±23	3207	± 10	23
TRBG-1-13.1	0.14	187	205	1.13	100	0.26720	0.66	22.89	1.4	0.6214	1.2	0.88	3116	±29	3289	± 10	5
TRBG-1-14.1	1.78	336	88	0.27	139	0.26012	0.62	16.93	1.7	0.4713	1.6	0.94	2489	±33	3250	± 9	23
TRBG-1-15.1	0.07	218	115	0.54	128	0.28513	0.38	26.80	1.2	0.6818	1.2	0.95	3351	±30	3391	± 6	1

Spot	$^{206}Pb_c$ (%)	U (ppm)	Th (ppm)	Th /U	$^{206}Pb^*$ (ppm)	$^{207}Pb^*$ $/^{206}Pb^*$	±%	$^{207}Pb^*$ $/^{235}U$	±%	$^{206}Pb^*$ $/^{238}U$	±%	err corr	$^{206}Pb/^{238}U$ Age (Ma)		$^{207}Pb/^{206}Pb$ Age (Ma)		Discordance (%)
TRG-2-1.1	0.18	70	17	0.25	41	0.28261	0.73	26.44	1.7	0.6790	1.5	0.90	3339	±39	3377	±11	1
TRG-2-1.2	0.02	298	176	0.61	170	0.26672	0.39	24.33	1.2	0.6617	1.1	0.94	3274	±29	3287	± 6	0
TRG-2-2.1	5.43	142	78	0.57	59	0.25511	2.10	15.88	2.5	0.4492	1.5	0.60	2392	±29	3224	±31	26
TRG-2-2.2	2.92	1021	145	0.15	192	0.18034	0.97	5.28	1.4	0.2123	1.0	0.74	1241	±12	2657	±16	53
TRG-2-3.1	0.10	278	208	0.77	145	0.25992	0.40	21.79	1.3	0.6080	1.2	0.95	3062	±29	3246	± 6	6
TRG-2-5.1	0.07	268	176	0.68	153	0.26651	0.39	24.46	1.2	0.6658	1.1	0.95	3289	±29	3285	± 6	0
TRG-2-6.1	0.07	225	215	0.99	127	0.26520	0.41	24.04	1.2	0.6574	1.2	0.94	3257	±30	3278	± 7	1
TRG-2-7.1	0.20	281	150	0.55	144	0.26184	0.42	21.41	1.2	0.5930	1.1	0.94	3002	±27	3258	± 7	8
TRG-2-8.1	0.10	430	357	0.86	194	0.24249	0.36	17.54	1.1	0.5244	1.1	0.95	2718	±24	3137	± 6	13
TRG-2-9.1	0.06	193	109	0.58	109	0.26748	0.45	24.16	1.3	0.6550	1.2	0.93	3248	±30	3291	± 7	1
TRG-2-10.1	0.10	270	164	0.63	148	0.28071	0.47	24.65	1.2	0.6368	1.1	0.92	3177	±28	3367	± 7	6
TRG-2-11.1	0.79	460	253	0.57	154	0.22832	0.50	12.16	1.2	0.3859	1.1	0.91	2104	±19	3041	± 8	31
TRG-2-12.1	1.79	355	297	0.86	156	0.24334	0.72	16.79	1.3	0.4999	1.1	0.85	2613	±24	3144	±11	17
TRG-2-13.1	0.10	470	389	0.85	183	0.24806	0.40	15.50	1.1	0.4532	1.1	0.94	2410	±21	3173	± 6	24
TRG-2-14.1	0.21	225	120	0.55	127	0.26701	0.44	24.13	1.2	0.6557	1.2	0.94	3250	±30	3288	± 7	1
TRG-2-15.1	0.10	408	220	0.56	185	0.26086	0.35	19.00	1.1	0.5282	1.1	0.95	2734	±24	3252	± 6	16
TRG-2-16.1	0.12	209	141	0.70	115	0.26280	0.43	23.11	1.2	0.6377	1.2	0.94	3180	±29	3264	± 7	3
TRG-2-17.1	4.27	255	214	0.87	90	0.25511	1.10	13.73	1.6	0.3891	1.2	0.76	2119	±21	3222	±16	34

Supplimentary Table. S2: Lu-Hf isotopic data of zircons from samples RM-1, RM-5, TRBG-1 and TRG-2 from Singhbhum Craton, Eastern India.

Sample	T(Ma)	Discorda-nce (%)	$^{176}Lu/^{177}Hf$ (p)	2 σ err	$^{176}Hf/^{177}Hf$ (p)	2 σ err	$^{176}Hf/^{177}Hf$ (t)	2 σ err	εHf(t)	± 2σ err
RM-1-1-1	3315 ± 8	17	0.00116	0.00000	0.28057	0.00003	0.28050	0.00007	-5.0	1.2
RM-1-2-1	3353 ± 13	17	0.00092	0.00000	0.28058	0.00003	0.28052	0.00007	-3.3	1.2
RM-1-3-1	4031 ± 5	0	0.00071	0.00000	0.28010	0.00004	0.28005	0.00007	-4.1	1.3
RM-1-3-2	4036 ± 15	1	0.00058	0.00001	0.28005	0.00004	0.28001	0.00007	-5.2	1.3
RM-1-4-1	3288 ± 16	24	0.00343	0.00005	0.28086	0.00005	0.28064	0.00010	-0.5	1.7
RM-1-5-1	3368 ± 12	11	0.00054	0.00001	0.28059	0.00004	0.28055	0.00007	-1.8	1.3
RM-1-6-1	2990 ± 8	36	0.00074	0.00000	0.28055	0.00004	0.28051	0.00007	-12.4	1.3
RM-1-7-1	3364 ± 7	4	0.00131	0.00001	0.28057	0.00004	0.28049	0.00007	-4.3	1.3
RM-1-8-1	2388 ± 180	52	0.00180	0.00002	0.28063	0.00004	0.28055	0.00007	-24.9	1.3
RM-1-9-1	3171 ± 8	25	0.00129	0.00005	0.28060	0.00004	0.28052	0.00008	-7.8	1.3
RM-1-10-1	3293 ± 17	14	0.00115	0.00001	0.28055	0.00004	0.28048	0.00007	-6.4	1.2
RM-1-11-1	2982 ± 21	42	0.00184	0.00006	0.28057	0.00004	0.28046	0.00007	-14.2	1.2
RM-1-12-1	3476 ± 6	20	0.00192	0.00005	0.28050	0.00004	0.28037	0.00007	-5.7	1.3
RM-1-13-1	3244 ± 21	38	0.00152	0.00002	0.28065	0.00004	0.28056	0.00008	-4.6	1.4
RM-1-14-1	3402 ± 8	-1	0.00040	0.00000	0.28057	0.00004	0.28055	0.00008	-1.3	1.4
RM-1-15-1	3330 ± 23	8	0.00172	0.00007	0.28064	0.00004	0.28053	0.00008	-3.6	1.3
RM-1-16-1	2091 ± 140	48	0.00188	0.00006	0.28057	0.00004	0.28050	0.00007	-33.7	1.3
RM-1-17-1	3084 ± 16	35	0.00204	0.00005	0.28064	0.00004	0.28052	0.00008	-9.7	1.4
RM-1-18-1	3108 ± 18	32	0.00137	0.00003	0.28065	0.00004	0.28056	0.00007	-7.6	1.3
RM-1-19-1	3362 ± 10	8	0.00199	0.00002	0.28060	0.00004	0.28047	0.00008	-4.9	1.5
RM-1-20-1	3308 ± 22	27	0.00080	0.00002	0.28060	0.00004	0.28055	0.00007	-3.5	1.3
RM-1-21-1	3365 ± 8	12	0.00490	0.00019	0.28090	0.00005	0.28059	0.00007	-0.8	1.3
RM-1-22-1	3385 ± 6	2	0.00100	0.00000	0.28058	0.00004	0.28052	0.00007	-2.8	1.3
RM-1-23-1	3400 ± 10	2	0.00138	0.00001	0.28058	0.00004	0.28049	0.00008	-3.2	1.4
RM-5-1-1	3396 ± 8	-3	0.00101	0.00001	0.28064	0.00003	0.28057	0.00007	-0.4	1.2
RM-5-2-1	3670 ± 7	5	0.00061	0.00004	0.28026	0.00003	0.28022	0.00006	-6.6	1.1
RM-5-3-1	3363 ± 11	0	0.00066	0.00001	0.28058	0.00004	0.28054	0.00007	-2.5	1.3
RM-5-4-1	3167 ± 8	26	0.00093	0.00001	0.28059	0.00004	0.28053	0.00008	-7.3	1.4
RM-5-5-1	3341 ± 5	10	0.00064	0.00000	0.28057	0.00004	0.28053	0.00008	-3.4	1.5
RM-5-6-1	3415 ± 7	-1	0.00063	0.00002	0.28051	0.00005	0.28047	0.00010	-3.7	1.8
RM-5-7-1	3071 ± 11	33	0.00106	0.00000	0.28058	0.00003	0.28051	0.00006	-10.3	1.1
RM-5-8-1	3386 ± 8	15	0.00114	0.00001	0.28062	0.00004	0.28054	0.00007	-1.9	1.3
RM-5-9-1	3673 ± 7	0	0.00105	0.00001	0.28034	0.00004	0.28027	0.00008	-4.7	1.4
RM-5-10-1	3396 ± 6	-1	0.00096	0.00001	0.28059	0.00004	0.28053	0.00008	-2.0	1.4
RM-5-11-1	4241 ± 4	-1	0.00125	0.00000	0.28005	0.00004	0.27995	0.00009	-2.5	1.6
RM-5-11-2	3858 ± 5	25	0.00192	0.00000	0.28009	0.00004	0.27994	0.00008	-11.9	1.5
RM-5-12-1	3390 ± 11	-1	0.00075	0.00000	0.28061	0.00003	0.28056	0.00007	-1.0	1.2
RM-5-13-1	3147 ± 14	33	0.00139	0.00001	0.28056	0.00003	0.28048	0.00006	-9.7	1.1
RM-5-14-1	3433 ± 6	-1	0.00144	0.00000	0.28060	0.00004	0.28051	0.00008	-1.9	1.4
RM-5-15-1	3394 ± 8	0	0.00116	0.00002	0.28061	0.00003	0.28054	0.00006	-1.8	1.0
RM-5-16-1	3381 ± 14	-1	0.00054	0.00000	0.28058	0.00003	0.28054	0.00006	-1.9	1.1
RM-5-17-1	3066 ± 25	35	0.00180	0.00001	0.28065	0.00004	0.28054	0.00008	-9.4	1.4
RM-5-18-1	3394 ± 7	2	0.00080	0.00001	0.28056	0.00004	0.28051	0.00007	-2.8	1.3
RM-5-19-1	3595 ± 12	5	0.00126	0.00001	0.28050	0.00004	0.28041	0.00007	-1.5	1.3

Sample	T(Ma)	Discor-dance (%)	$^{176}Lu/^{177}Hf$ (p)	± 2σ err	$^{176}Hf/^{177}Hf$ (p)	± 2σ err	$^{176}Hf/^{177}Hf$ (t)	± 2σ err	εHf (t)	± 2σ err
TRBG-1-1-1	3352 ± 6	8	0.00093	0.00001	0.28066	0.00004	0.28060	0.00007	-0.7	1.3
TRBG-1-2-1	2905 ± 10	37	0.00098	0.00001	0.28067	0.00003	0.28061	0.00007	-10.8	1.2
TRBG-1-3-1	3361 ±7	-1	0.00098	0.00001	0.28075	0.00004	0.28068	0.00008	2.6	1.4
TRBG-1-4-1	2972 ± 19	42	0.00123	0.00002	0.28059	0.00005	0.28052	0.00009	-12.3	1.6
TRBG-1-4-2	1128 ± 420	33	0.00110	0.00002	0.28070	0.00004	0.28068	0.00008	-49.5	1.3
TRBG-1-5-1	3222 ± 8	20	0.00095	0.00006	0.28059	0.00005	0.28053	0.00009	-6.1	1.6
TRBG-1-6-1	3248 ± 12	11	0.00059	0.00001	0.28063	0.00004	0.28060	0.00008	-3.1	1.4
TRBG-1-7-1	3129 ± 20	31	0.00134	0.00004	0.28052	0.00004	0.28044	0.00008	-11.4	1.4
TRBG-1-7-2	1374 ± 300	41	0.00161	0.00002	0.28036	0.00006	0.28032	0.00011	-56.4	2.0
TRBG-1-8-1	3267 ± 6	10	0.00132	0.00001	0.28070	0.00004	0.28062	0.00007	-2.0	1.3
TRBG-1-9-1	3340 ± 6	8	0.00117	0.00002	0.28058	0.00004	0.28051	0.00007	-4.0	1.2
TRBG-1-10-1	3404 ± 7	2	0.00097	0.00003	0.28051	0.00004	0.28045	0.00007	-4.7	1.3
TRBG-1-11-1	3200 ± 10	25	0.00118	0.00001	0.28070	0.00003	0.28062	0.00007	-3.3	1.2
TRBG-1-12-1	3207 ± 10	23	0.00093	0.00001	0.28065	0.00004	0.28060	0.00007	-4.2	1.3
TRBG-1-13-1	3289 ± 10	5	0.00096	0.00003	0.28062	0.00004	0.28056	0.00007	-3.6	1.3
TRBG-1-14-1	3250 ± 9	23	0.00123	0.00003	0.28065	0.00003	0.28057	0.00006	-4.0	1.1
TRG-2-1-1	3377 ± 11	1	0.00104	0.00003	0.28065	0.00003	0.28058	0.00006	-0.6	1.1
TRG-2-1-2	3287 ± 6	0	0.00083	0.00001	0.28065	0.00003	0.28060	0.00006	-2.2	1.1
TRG-2-2-1	3224 ± 31	26	0.00325	0.00005	0.28075	0.00003	0.28054	0.00006	-5.6	1.1
TRG-2-2-2	2657 ± 16	53	0.00263	0.00003	0.28080	0.00004	0.28067	0.00007	-14.4	1.3
TRG-2-3-1	3246 ± 6	6	0.00170	0.00001	0.28073	0.00003	0.28063	0.00006	-2.1	1.2
TRG-2-5-1	3285 ± 6	0	0.00139	0.00000	0.28070	0.00003	0.28061	0.00006	-1.7	1.1
TRG-2-6-1	3278 ± 7	1	0.00168	0.00001	0.28070	0.00003	0.28060	0.00006	-2.5	1.1
TRG-2-7-1	3258 ± 7	8	0.00120	0.00001	0.28068	0.00003	0.28061	0.00007	-2.6	1.2
TRG-2-8-1	3137 ± 6	13	0.00249	0.00011	0.28073	0.00005	0.28058	0.00009	-6.4	1.6
TRG-2-9-1	3291 ± 7	1	0.00155	0.00001	0.28070	0.00005	0.28060	0.00009	-1.9	1.6
TRG-2-10-1	3367 ± 7	6	0.00057	0.00001	0.28063	0.00004	0.28060	0.00007	-0.3	1.3
TRG-2-11-1	3041 ± 8	31	0.00122	0.00002	0.28065	0.00004	0.28058	0.00008	-8.5	1.4
TRG-2-12-1	3144 ± 11	17	0.00190	0.00000	0.28073	0.00004	0.28062	0.00007	-4.8	1.3
TRG-2-13-1B	3173 ± 6	24	0.00118	0.00001	0.28060	0.00003	0.28052	0.00006	-7.6	1.1
TRG-2-14-1	3288 ± 7	1	0.00204	0.00002	0.28070	0.00003	0.28057	0.00007	-3.1	1.2
TRG-2-15-1	3252 ± 6	16	0.00148	0.00007	0.28062	0.00003	0.28053	0.00006	-5.4	1.0
TRG-2-16-1	3264 ± 7	3	0.00076	0.00001	0.28065	0.00003	0.28060	0.00007	-2.7	1.2
TRG-2-17-1	3222 ± 16	34	0.00160	0.00005	0.28071	0.00004	0.28061	0.00006	-3.3	1.2

*$^{176}Lu/^{177}Hf$ (p) and $^{176}Hf/^{177}Hf$ (p) denote present day isotopic ratios.

*$^{176}Hf/^{177}Hf$ (t) denotes initial isotopic ratio calculated using $^{207}Pb/^{206}Pb$ crystallization age (Ma) of the respective domain.
